# Expression of the dopaminergic D1 and D2 receptors in the anterior cingulate cortex in a model of neuropathic pain

**DOI:** 10.1186/1744-8069-7-97

**Published:** 2011-12-15

**Authors:** J Manuel Ortega-Legaspi, Patricia de Gortari, René Garduño-Gutiérrez, María Isabel Amaya, Martha León-Olea, Ulises Coffeen, Francisco Pellicer

**Affiliations:** 1 Laboratorio de Neurofisiología Integrativa. Dirección de Neurociencias. Instituto Nacional de Psiquiatría Ramón de la Fuente. México; 2 Laboratorio de Neurofisiología Molecular. Dirección de Neurociencias. Instituto Nacional de Psiquiatría Ramón de la Fuente. México; 3 Departamento de Neuromorfología Funcional. Dirección de Neurociencias. Instituto Nacional de Psiquiatría Ramón de la Fuente. México; 4 Emory University School of Medicine, Atlanta, GA. USA

## Abstract

**Background:**

The anterior cingulate cortex (ACC) has been related to the affective component of pain. Dopaminergic mesocortical circuits, including the ACC, are able to inhibit neuropathic nociception measured as autotomy behaviour. We determined the changes in dopamine D1 and D2 (D1R and D2R) receptor expression in the ACC (cg1 and cg2) in an animal model of neuropathic pain. The neuropathic group had noxious heat applied in the right hind paw followed 30 min. later by right sciatic denervation. Autotomy score (AS) was recorded for eight days and subsequently classified in low, medium and high AS groups. The control consisted of naïve animals.

A semiquantitative RT-PCR procedure was done to determine mRNA levels for D1R and D2R in cg1 and cg2, and protein levels were measured by Western Blot.

**Results:**

The results of D1R mRNA in cg1 showed a decrease in all groups. D2R mRNA levels in cg1 decreased in low AS and increased in medium and high AS. Regarding D1R in cg2, there was an increase in all groups. D2R expression levels in cg2 decreased in all groups. In cg1, the D2R mRNA correlated positively with autotomy behaviour. Protein levels of D2R in cg1 increased in all groups but to a higher degree in low AS. In cg2 D2R protein only decreased discretely. D1R protein was not found in either ACC region.

**Conclusions:**

This is the first evidence of an increase of inhibitory dopaminergic receptor (D2R) mRNA and protein in cg1 in correlation with nociceptive behaviour in a neuropathic model of pain in the rat.

## Background

The anterior cingulate cortex (ACC) is a structure that has been related to the affective component of pain [1,2]. Evidence obtained with functional imaging techniques has shown that the ACC is activated by pain in correlation with the unpleasantness of the stimulus [3,4]. Translating those findings into animal research, painful stimuli also elicit functional activation of this structure in rats [5,6]. Furthermore, the specific site within the ACC that is particularly related to central pain processing has been defined in humans as the pregenual part of the ACC [7] and as the rostral ACC in rats. Moreover, the rostral ACC, also known as cg1, has specifically been related to the affective component of pain in rodents [8] while more caudal regions are involved on motor planning as a secondary response to nociceptor stimulation [9].

This cortex receives dopaminergic input from the ventral tegmental area (VTA) via the medial forebrain bundle [2,10]. Dopaminergic mesocortical circuits, including the ACC, are able to inhibit neuropathic nociception measured as autotomy behaviour. In this context, electrical stimulation of the VTA diminishes self-injury behaviour in an inflammatory model of pain in rats [11]. Although the VTA projects monosynaptically to the ACC, its role is not limited to the ACC given that it also provides dopaminergic input to other areas of the cerebral cortex where it is an important inhibitor of nociception [12]. More specifically, there is electrophysiological evidence about the subdivisions of the ACC that are responsive to nociceptor stimulation in which cg1 (rostral) responds and cg2 does not [13].

There is evidence that when dopamine is microinjected into the ACC in an animal model of neuropathic pain, neurectomy-induced nociception measured as autotomy behaviour, is reduced [14]. Recent evidence shows that the systemic administration of amantadine, a known dopamine releaser [15], increases dopamine content in the ACC when measured using HPLC [16] and diminishes neuropathic nociception induced by denervation when administered both systemically (i.p.) [17] or microinjected into the ACC [14]. Moreover, there is evidence that activation of dopamine D1 receptors (D1R) enhances nociception while that of D2 receptors (D2R) decreases it when the insular cortex is manipulated [18]. Further evidence involving this model and neurotransmission system shows an increased incidence and acceleration of autotomy following chemical ablation of dopaminergic terminals in the striatum or selective ablation (6-OHDA) of dopaminergic neurons in the *substantia nigra *and VTA [19,20].

Even though it is known that dopamine can modulate the functions of the ACC and that this is a major site of nociceptive modulation, there is little evidence about the expression of dopaminergic receptors during neuropathic nociception.

In this study, we performed a semiquantitative RT-PCR procedure to evaluate changes in the mRNA levels of D1R and D2R in the ACC of rats that were subjected to neuropathic nociception induced by denervation and in naïve controls. Also, using the same model, we quantified protein levels of these receptors using a Western blot technique. The animal model used in this work has been widely used as an experimental model of phantom limb and neuropathic pain by several groups [21-24].

## Results

### D1R and D2R mRNA changes

**Figure 1:**
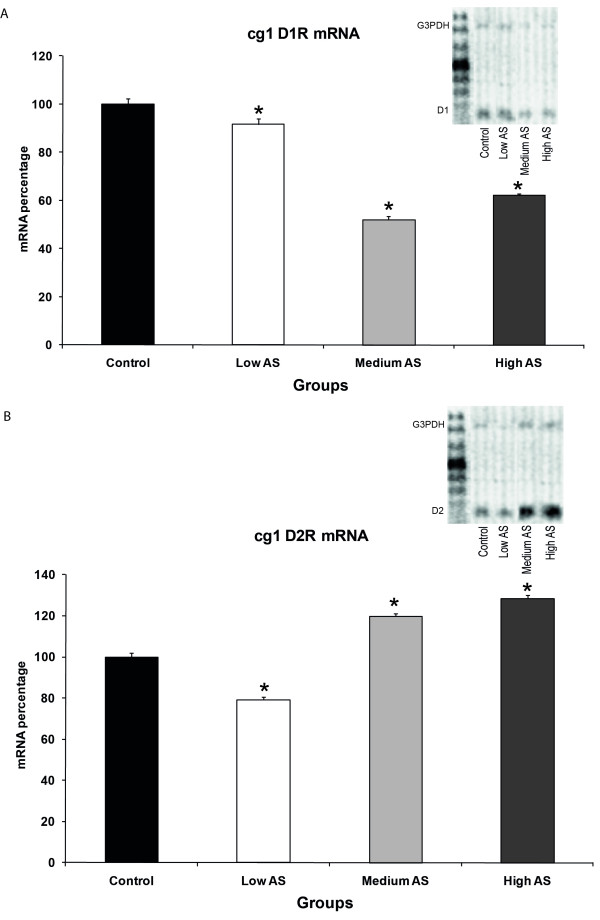
**A. Semiquantitative mRNA levels for D1R in cg1**. The graph depicts a decrease in all groups when compared to control. Low, medium and high AS groups show 92 ± 2.4, 52 ± 1.3 and 62 ± 0.6%, respectively considering the control group as 100%. The lowest mRNA level is in the medium AS group. Image B shows mRNA levels for D2R in the same region with 79 ± 1.2, 120 ± 1.5 and 129 ± 1.6% for groups with low, medium and high AS, respectively. Notice that there is a progressive increase in mRNA levels, as the autotomy behaviour increases (n = 4 in all groups). All percentages are significantly different when compared to control (one-way ANOVA, Bonferroni post hoc *p < 0.05). At the top of each frame, there is an example of the electrophoresis agarose gel showing the control oligonucleotide (upper band) and the cDNA of D1R's in (A) and D2R's in (B) (lower band).

**Figure 2:**
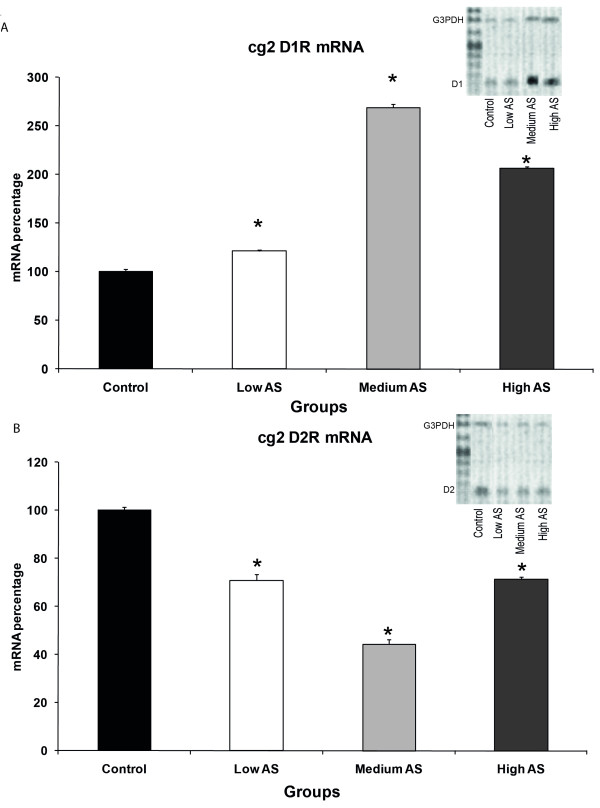
**A. This figure shows semiquantitative mRNA levels for D1R in cg2**. Notice an increase in all groups when compared to control. Low, medium and high AS groups show 121 ± 1.1, 269 ± 3.3 and 207 ± 1%, respectively considering the control group as 100%. The greatest increase is in the medium AS group. Image B shows mRNA levels for D2R in the same region with 71 ± 2.6, 44 ± 1.8 and 71 ± 0.8% for groups with low, medium and high AS, respectively. Also, notice that the lowest value is for the medium AS group (n = 4 in all groups). All percentages are significantly different when compared to control (one-way ANOVA, Bonferroni post hoc *p < 0.05). At the top of each frame, there is an example of the electrophoresis agarose gel showing the control oligonucleotide (upper band) and the cDNA of D1R's in (A) and D2R's in (B) (lower band).

The results showed a differential expression of dopamine receptors D1 and D2 in cg1 and cg2 depending on the degree of nociception measured as autotomy behaviour. In cg1, there was a decrease in D1R mRNA in all AS groups (92 ± 2.4% for low, 52 ± 1.3% for medium and 62 ± 0.6% for high AS groups, one-way ANOVA F = 161.853, p = 0.001. Figure 1A) with the greatest one in the medium AS group. D2R mRNA decreased in the low AS group (79 ± 1.2%) and progressively increased in medium (120 ± 1.5%) and high AS (129 ± 1.6%) groups (one-way ANOVA F = 192.597, p = 0.001. Figure 1B). Regarding cg2, D1R mRNA showed an increase in all AS groups (121 ± 1.1% for low, 269 ± 3.3% for medium and 207 ± 1% for high AS groups, one-way ANOVA, F = 1181.367, p = 0.001. Figure 2A) with the greatest one in the medium AS group. As for cg2, D2R mRNA content depicted a decrease in all groups (71 ± 2.6% for low, 44 ± 1.8% for medium and 71 ± 0.8% for high AS groups, one-way ANOVA, F = 159.104, p = 0.001) which was greater in the medium AS group (Figure 2B). All the previous results were statistically significant when compared to controls (one-way ANOVA, Bonferroni *post hoc *p < 0.05).

### D1R and D2R protein changes

**Figure 3:**
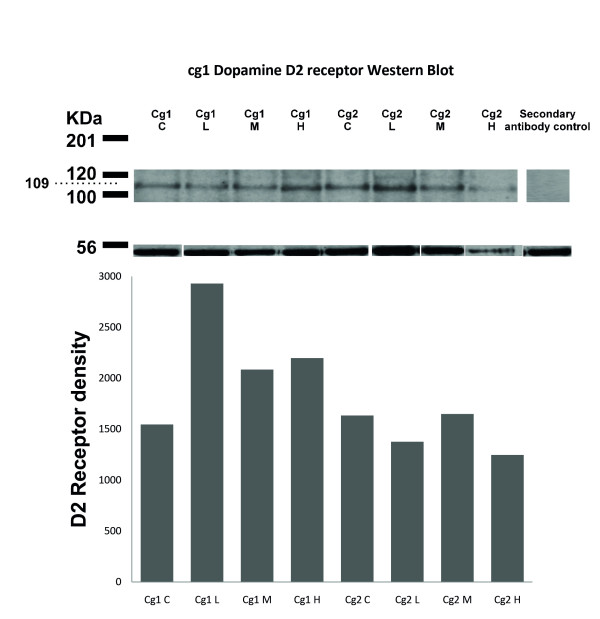
**Western Blot results of D2Rin ACC cg1 and cg2 regions**. The bars represent the area between the optical density (in px^2^) of D2 and actin shown in the gel above. The different experimental groups for each region were L (Low AS), M (Medium AS) and H (High AS). In cg1 there is an important increase (90%) in the expression of the receptor in the low AS group when compared to medium AS (35%) and high AS (42%). Cg2 showed 16, 1 and 24% decrease in the groups low AS, medium AS and high AS respectively.

The results showed that D2R have a higher expression as protein in cg1 in rats with low AS when compared to controls and rats with medium and high AS. The group with low AS showed a 90% increase (2930 pixel squared, px^2^) in expression when compared to control (100% or 1545 px^2^) whereas the medium and high AS groups showed an increase of 35% (2085 px^2^) and 42% (2198 px^2^) respectively. In cg2, protein levels of this receptor showed a 16% (1377 px^2^) decrease in the low AS group, an increase of 1% (1649 px^2^) in the medium AS one and a 24% (1247 px^2^) decrease in the high AS group compared to control (1634 px^2^. Figure 3).

**Figure 4:**
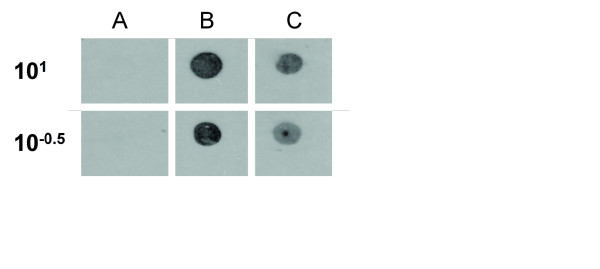
**Immuno Dot performed for D1R in control animals in the cerebral cortex**. This control was done in order to validate the experimental procedure for dopamine D1 receptors in the ACC and the specificity of the antibody. The (A) column shows the blocking D1R peptide without primary antibody, (B) shows the blocking D1R peptide with D1R antibody and (C) the result in the cerebral cortex of control animals. The concentrations shown are the ones with positivity; the results were negative from 10^-3^.

D1R were not expressed in either cg1 or cg2 under the circumstances studied. In order to evaluate the efficacy of the experimental conditions, these were tested in a control group in the caudoputamen (Western Blot, data not shown) and cerebral cortex (Immuno Dot). The results in these controls showed an expression of D1R and a validation of the experimental conditions used (Figure 4).

## Discussion

Several authors have described dopaminergic neurones whose soma reside in the VTA and project to the ACC, which is part of the pain matrix [16,25,26]. The results in this work showed that peripheral nerve lesions trigger changes in the dynamics of central dopaminergic receptors within the pain matrix. These changes would explain the development and consequences of pain states, *ie*. hyperalgesia, allodynia, phantom limb pain, among others.

The results showed that animals under neuropathic nociception induced by noxious heat and a sciatic neurectomy have an increased expression of mRNA levels of dopamine D2 receptors in cg1 compared to naïve animals. Interestingly, protein levels, which represent the functional state of the receptors, did not increase as much with higher autotomy scores. This suggests that the expression of D2R is partially responsible for the development of autotomy behaviour. Also, the increase in D2R mRNA allows us to infer that there is a counter-regulatory mechanism within cg1 that would increase inhibitory receptors. Electrophysiological evidence showed that cg2 is not a nociceptive target whereas cg1 indeed is [13].

In respect to dopamine D2 receptor mRNA and protein mismatch, the regulation of these has been shown not to occur in parallel when modified by pharmacological stimuli [27]. A mismatch in mRNA and protein synthesis in the CNS has been previously described [28]. A possible explanation of this would be given by the prior utilization of mRNA in order to produce the protein as seen in the results of the present work.

The behavioural expression of pain in animals has been studied at a genetic level [29,30] to the point that we now have high and low autotomy strains. Interestingly enough, the sample of animals used in this study was random which still showed that a difference in the expression of a receptor has an impact in the degree of autotomy. Furthermore, while this behaviour is developed over time, the present study shows a snapshot of the average day (eight) in which a control animal reaches its AS mean [14,18,31].

Dopamine receptors mRNA expression in cg2 is opposite to that of cg1, which shows an increase of D1R and a decrease in D2R mRNA levels. Furthermore, protein levels of D2R in cg2 vary only discretely when compared to cg1. These data suggest that dopaminergic receptors have differential physiology in terms of its anatomical localisation.

In regard of dopamine D1 receptors, there was a decreased mRNA expression in cg1 and an increased one in cg2. The results did not show a correlation with AS in either region. Interestingly, under the experimental conditions used, we did not find an expression of D1R protein in either region. We validated the experiment by measuring this receptor in the caudoputamen and the specificity was also validated by an Immuno Dot in the cerebral cortex. With this we were able to see that D1R plays a more discrete role in the modulation of neuropathic pain in the ACC in rodents as compared to D2R. Even though D1R was not evident as protein, the pharmacological blockade of D1R modifies pain related behaviour in the insular cortex which is also part of the pain matrix [18]. In spite of these results, we cannot fully discard the role of D1R's in the modulation of chronic neuropathic pain.

The role of D2R's has also been studied at a pharmacological and neurochemical level. The selective activation of D2R in the rostral agranular insular cortex diminishes nociception in the model used in the present work [18]. This result has recently been replicated in other structures that belong to the so called 'pain matrix', such as the ventrolateral orbital cortex [32], the nucleus accumbens [33,34] and the dorsolateral striatum [35].

Receptor expression regulation of pain related behaviours and mechanisms seems to behave somehow in an analogous way as intracellular control pathways. A clear example of this is the regulation of NMDA receptor, which is well known to play an important role in the modulation of pain, particularly in the ACC [36]. This receptor activates transduction pathways like those of PKA and ERK that when inhibited decrease synaptic plasticity in an arthritis pain model [37]. Dopaminergic receptors have intracellular mediators that can potentially play a role in the findings hereby presented, like the fragile × mental retardation protein which is a key messenger for dopamine modulation in the forebrain [38].

The receptor dynamic in which the mRNA of an inhibitory receptor increases when the protein levels are lower seems to be similar with different neurotransmission systems (muscarinic) in the same model of neuropathic pain [39]. In regard of the dopaminergic system, animals under inflammatory pain show a similar pattern of dopamine D1 and D2 receptor mRNA expression in the insular cortex [40]. In another experimental approach with the same neurotransmission system, the increase of D2R in the nucleus accumbens decreases addiction-related behaviours [41,42]. This supports that there is a general mechanism of central control of different basic behaviours, structures and neurotransmission systems.

## Conclusions

To our knowledge, this is the first evidence of changes in the expression of an inhibitory dopaminergic D2R in a *locus *that is well known to play a key role in the processing of nociceptive input (cg1) in correlation with pain related behaviour in a neuropathic model of pain in the rat.

## Materials and methods

The experiments were conducted in agreement with the ethics committee regulations of the International Association for the Study of Pain [43] and with the project's commission approval of the Instituto Nacional de Psiquiatría Ramón de la Fuente (INPRF). Male Wistar rats (250-350 g) were raised, housed and maintained in the INPRF's animal house. During the observation period the animals were maintained in transparent acrylic individual cages with light-dark cycles of 12 × 12 h, with *ad libitum *feeding and hydration.

### Neuropathic pain model

We used the neuropathic pain model induced by denervation [23]. This experimental approach triggers a quantifiable behaviour known as autotomy that is related to the degree of neuropathic pain [22]. In order to enhance autotomy and shorten its onset, noxious heat was applied prior to denervation [44]. Briefly, a nociceptive process was induced by immersing the rat's right hind paw in hot water at 55°C for 20 s, 30 min prior to denervation. The right sciatic nerve was exposed by microdissection. The nerve was cut and ligated with silk 3-0 suture. Five millimetres of the distal end were removed in order to avoid reinnervation. Skin was closed with silk 3-0 suture. Animals were anaesthetised with 2% isofluorane throughout these procedures. The wounds were checked every day for possible signs of infection and behaviour of rats was compared with that of the controls for the remainder of the experiments with no noticeable differences.

### Autotomy behaviour measurement

Daily autotomy scores (AS) were computed using a modified scale devised by Wall et al. (1979). This scale gives the following scores: 1 point for the removal of one or more nails; 1 additional point for each distal half digit attacked and a further point for each proximal half digit attacked. If the distal or proximal half of the paw was attacked an additional point of 1 is added for each one. AS was recorded for eight days which has proven to be the average day in which AS reaches its media in control rats from previous studies, [14,18,31]. Then, these groups were divided into low, medium and high AS groups. This categorisation was done considering the lowest (AS from 0 to 1) and the highest (AS from 11 to 13) autotomy scores. Those animals whose AS fell in the middle were categorised as medium AS. We separated the animals according to AS in order to evaluate the changes related to those animals according to the lowest and greatest pain as well as those with medium AS values. This model has proven to be a useful tool to study central mechanisms of neuropathic pain [22]. Moreover, the behaviour associated with autotomy has been studied to a genetic level in which high and low pain phenotype strains have been produced [29,30]. In this work, we used a random selection of animals and categorised them according to their autotomy score. Since autotomy is related to the intensity of neuropathic pain-like behaviour, we wanted to know if D1R and D2R mRNA and protein expression in the ACC is correlated with AS.

### Tissue extraction

**Figure 5:**
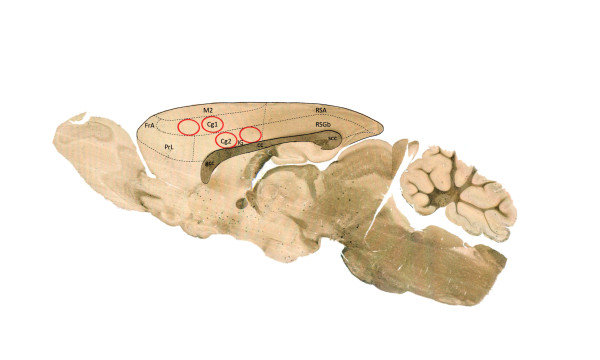
**The circles in red represent the *loci *in which the tissue was extracted for RT-PCR and Western Blot**. Notice that cg1 corresponds to the rostral part of the ACC and cg2 to the ventral one. Modified from Paxinos and Watson, 1998 [45].

On day eight, all animals were sacrificed by decapitation and their brain was extracted and frozen in dry ice (-70°C). In order to extract the left ACC (contralateral to denervated hind paw), a parasagital brain slice was obtained by cutting the frozen brain longitudinally at 1 mm to the left from the midline. Then, two punches with a 1.0 mm diameter sample corer were done in cg1 and in cg2 (Figure 5) [45]. The *corpus callosum *was used as anatomical reference.

### Reverse transcriptase polymerase chain reaction (RT-PCR) procedure

mRNA semi-quantification, by RT-PCR: Frozen cg1 and cg2 were homogenized in 4 M guanidine thyocianate (ICN, Aurora, Ohio, USA) and total RNA extracted as has previously been described [46]. RNA quality of samples was verified by the ratio of O.D. absorbencies 260/280 nm and 260/230 nm considered appropriate when value was >1.8, and by electrophoresis quantifying 28S/18S ratio and discarded if lower than 1.8 or, when evidence of degradation was observed by increased staining at the end of the gel. mRNA levels of D1R and D2R from cg1 and cg2 were semi-quantified by reverse-transcriptase polymerase chain reaction (RT-PCR), using glyceraldehyde 3-phosphate dehydrogenase (G_3_PDH) as control transcripts. The protocol used was essentially as described in [47]: 1.5 μg of RNA was used to obtain cDNA (M-MLV reverse transcriptase (Carlsbad, CA, USA) and oligo-dT (Universidad Nacional Autónoma de México UNAM Biotechnology Institute's facilities), followed by the PCR reaction: the number of cycles for each probe was optimized for each region to assure linear conditions, using 1 μl and 10 pmol of D1R probe (sense sequence: CAT TCT GAA CCT CTG CGT GA; antisense: GTT GTC ATC CTC GGT GTC CT), for D2R using 1 μl and 25 pmol (sense sequence: CAT TGT CTG GGT CCT GTC CT; antisense: GAC CAG CAG AGT GAC GAT GA); and 1 μl and 50 pmol G_3_PDH (sense sequence: TGA AGG TCG GTG TCA ACG GAT TTG GC; antisense: CAT GTA GGC CAT GAG GTC CAC CAC) and 0.5 ml Taq DNA polymerase (5U/ml) (Biotecnologías Universitarias, UNAM, DF, México). Oligonucleotides were synthesized at the Instituto de Biotecnología, UNAM. Final conditions for cg1 and cg2 were: 30 cycles for D1R and D2R and 21 for G_3_PDH. Each cycle consisted of 95°C for 1 min followed by: 1 min at 64°C for D1R, D2R and G_3_PDH; all followed by 1 min 15 s at 72 °C. All cDNAs had a final extension of 10 min at 72 °C. Several cDNAs were semiquantified from the same RT reaction.

RT-PCR products (10 μl of each DNA, and 5 μl of G_3_PDH) were separated by 2% of agarose (Ultra-pure Bio-Rad, Hercules CA, USA) gel electrophoresis, stained with ethidium bromide (1 mg/L) and density measured with the Advanced American Biotech Imaging software (American-Applied Biotechnology, Fullerton, CA, USA). The relative amounts of the studied cDNAs were calculated as the ratio of each cDNA over G_3_PDH densities. Care was taken to include samples of controls and experimental groups in the same gel.

### Western Blot Procedure

Tissue was homogenized at 4°C with a Teflon glass pestle in a buffer solution containing 250 mM sucrose, 1 mM EDTA, 10 mM Tris (pH 7.2) and one tablet/40 ml of complete protease inhibitor cocktail (Boehringer Mannheim). The homogenate was centrifuged at 3000 × g for 15 min at 4°C. The supernatant was centrifuged at 20,000 × g for 30 min at 4°C. Subsequently, the supernatant was centrifuged at 100,000 × g for 45 min at 4°C. The pellet was re-suspended in the homogenization buffer containing protease inhibitors and stored at -80 °C until used for electrophoresis. Protein concentration was determined using a Micro-BCA protein assay kit (Pierce, cat. 23235, Rockford, IL, USA).

For electrophoresis, pre-stained molecular weight markers (SDS±Polyacrylamide Gel Electrophoresis Broad Range, Cat. 161-0318 Bio-Rad, Hercules, CA, USA) and membrane protein samples were diluted with the 2 × electrophoresis sample buffer, boiled for 10 min and cooled at room temperature. Denatured protein samples were then separated on a discontinuous (4 ± 7.5%) SDS±polyacrylamide Laemmli gel system by means of a Bio-Rad Miniprotean II cell device for 1 h at 90, 120V respectively. For western blotting, proteins were electrophoretically transferred from resolving gels to nitrocellulose membranes (0.2 μm, Bio-Rad) in transfer buffer [192 mM glycine, 25 mM Tris±HCl (pH 8.3) and 20% methanol] for 1 h at 100 V using a Bio-Rad Trans-blot tank apparatus at 48C. Blotted membranes were rinsed twice with PB 10 mM plus 0.09% NaCl (pH 7.4), and blocked for 1 h at room temperature with a solution containing PB 10 mM plus 0.09% NaCl (pH 7.4))/0.3% Tween-20 (Bio Rad, cat 170-6531), 3% teleostean gelatin (Sigma, cat. G7765) and 0.3% milk (svelty non fat) all purchased from Bio Rad. Membrane blots were incubated overnight at 4°C plus 1 hr at room temperature with Rabbit anti D2R, (Chemicon, cat AB5084P, dil 1:100) and Goat anti actin, (Santa cruz, cat, SC-1616, dil 1:8333) in the blocking solution. After four 5-min washes with 0.2% Tween-20, 3% Gelatin in PB 10 mM plus 0.09% NaCl (pH 7.4) solution, the membrane was incubated with secondary antibodies HRP donkey anti Rabbit (Jackson immunoresearch, cat. 711-035-152, dil 1:5000) and HRP donkey anti goat (Sta. Cruz, cat. SC-2020, 1:10000), in 0.3% Tween 20, 3% Gelatin, teleostan, 0.3% milk svelty for the HRP donkey anti rabbit and 3.5% for HRP donkey anti goat, for 2 h at room temperature. After four washes in PB 10 mM plus 0.09% NaCl/0.1% Tween-20 at room temperature, bound antibody was visualized on films (Kodak, cat 604-0331) using an enhanced chemiluminescence kit (western lighting chemiluminescence Reagent Plus NEN, cat. NEL 105001EA; Waltham, MA USA).

### Immuno Dot procedure

In order to validate the specificity of D1R antibodies, an immuno dot was performed in the cerebral cortex of control animals. Briefly, a 0.22 μm pore nitrocellulose membrane was marked into squares of 1x1 cm. Then, it was activated with 20% MetOH/PB 10 mM pH 7.2 low salt (0.09% NaCl) for 15 min. and allowed to dry at room temperature. Once activated, 5 mL of each membrane sample were placed in a table (previously dissolved in 20% MetOH/PB low salt) in the different working concentrations (10^1^, 10^-0.5 ^and 10^-3^). Excess MetOH in descending order of concentration was removed, 10% MetOH/PB and a washed in low salt with PB only, 5 min. Each wash was done while being shacked with CTE at room temperature. The membrane was preblocked with a solution of 0.3% T-20 (Tween 20 BIORAD, Cat. 170-6531), 3% gelatin (Gelatin teleost, SIGMA, cat. G-7765), 0.8, 3% milk Svelty in PB low salt (0.09% NaCl) 10 mM, pH 7.2, for 1-1 1/2 hr, with stirring CTE at T ° A. Then, the membranes were incubated in the primary Ab Goat anti D1R (Santa Cruz, cat. sc-1434) dil. 1:5000 in the same solution at 4 ° C overnight with stirring CTE. After the incubation time, the membranes remained for an additional hour at room temperature. After this, four washes with 0.2% T-20, 3% gelatin in PB low salt for 10 min. with shaking CTE, were performed. The secondary antibody was incubated, HRP Donkey anti goat (Santa Cruz, sc-2020) dil 1:10,000 in 0.3% T-20, 3% gelatin, 1% milk Svelty, in 10 mM, PB low salt, pH 7.2, for 2 hr, with stirring CTE at room temperature followed by two washes with 0.1% T-20/PB low salt for10 min. each. Two final washes with low salt PB were done afterwards. Finally, complex chemiluminiscense (Perkin Elmer, cat. NEN 101) was added and revealed in an ultrasensitive x-ray film (Kodak X-Omat).

### Experimental groups

- Control (n = 12): animals that were maintained in individual cages for 8 days with no surgical interventions. Four animals were used for the RT-PCR experiments and eight for the analysis of protein levels with Western blot.

- Neuropathic group (n = 40): all the animals had noxious heat applied to the right hind paw followed by a right sciatic denervation 30 minutes later. AS was recorded for eight days and after that period, the animals were divided in three different AS groups: low, medium, and high. To perform RT-PCR experiments, four animals per group were used [48]. For the Western Blot experiments 10 rats were used for the low AS group, 13 for medium AS and 5 for high AS.

Differences in D1 and D2 receptors mRNA levels between groups were analysed by a one-way ANOVA followed by a *post hoc *Bonferroni test was used. Significance was considered when p < 0.05.

The RT-PCR and Western Blot procedures were carried out in a blinded fashion.

## Competing interests

The authors declare that they have no competing interests.

## Authors' contributions

JMOL: participated in the design of the study and drafted the manuscript; PG: molecular RT-PCR procedures; RG: western Blot and immuno dot procedure; MIA: RT-PCR determinations; ML: western Blot and immuno dot procedures; UC: performed the statistical analysis and drafted the manuscript; FP: conceived of the study, design, coordination and drafted the manuscript. All authors read and approved the final manuscript.
